# Long-term Administration of Nuclear Bile Acid Receptor FXR Agonist Prevents Spontaneous Hepatocarcinogenesis in Abcb4^−/−^ Mice

**DOI:** 10.1038/s41598-017-11549-7

**Published:** 2017-09-11

**Authors:** Marica Cariello, Claudia Peres, Roberta Zerlotin, Emanuele Porru, Carlo Sabbà, Aldo Roda, Antonio Moschetta

**Affiliations:** 1 Department of Interdisciplinary Medicine, “Aldo Moro” University of Bari, 70124 Bari, Italy; 2 INBB, National Institute for Biostructures and Biosystems, 00136 Rome, Italy; 3 National Cancer Research Center, IRCCS Istituto Tumori “Giovanni Paolo II”, 70124 Bari, Italy; 4 Department of Chemistry “Giacomo Ciamician”, University of Bologna, 40126 Bologna, Italy

## Abstract

Altered bile acid (BA) signaling is associated with hepatotoxicity. The farnesoid X receptor (FXR) is a nuclear receptor that transcriptionally regulates BA homeostasis. Mice with FXR ablation present hepatocarcinoma (HCC) due to high toxic BA levels. Mice with Abcb4 ablation accumulate toxic BA within the bile ducts and present HCC. We have previously shown that intestinal specific activation of FXR by transgenic VP16-FXR chimera is able to reduce BA pool size and prevent HCC. Here we tested chemical FXR activation by administering for 15 months the dual FXR/ membrane G protein-coupled receptor (TGR5) agonist INT-767 (6α-ethyl-3α,7α,23-trihydroxy-24-nor-5β-cholan-23-sulphate) to Fxr^−/−^ and Abcb4^−/−^ mice. HCC number and size were significantly reduced by INT-767 administration. In contrast, no changes in HCC tumor number and size were observed in Fxr^−/−^ mice fed with or without INT-767. Notably, INT-767 preserved the hepatic parenchyma, improved hepatic function and down-regulated pro-inflammatory cytokines. Moreover, in Abcb4^−/−^ mice INT-767 prevented fibrosis by reducing collagen expression and deposition. Thus, long term activation of FXR is able to reduce BA pool, reprogram BA metabolism and prevent HCC. These data provide the impetus to address the bona fide therapeutic potential of FXR activation in disease with BA-associated development of HCC.

## Introduction

Bile acids (BAs) are the end products of cholesterol catabolism, synthesized in the liver and released into the small intestine after meal ingestion. BAs facilitate the intestinal digestion and absorption of dietary fat, steroids, drugs and lipophilic vitamins. Most BAs are reabsorbed in the ileum and transported back to the liver for re-secretion into the gallbladder^1, 2^. Altered BA signaling in the liver and intestine is associated with severe disease including the development of cholestasis and hepatocellular carcinoma (HCC)^3, 4^.

BA production and circulation are tightly regulated *via* the nuclear receptor, farnesoid X receptor (FXR)^5, 6^. In the liver, FXR reduces conversion of cholesterol to BAs by downregulating the rate limiting enzyme of BA synthesis cytochrome P450 A1 (CYP7A1), *via* the small heterodimer partner (SHP)^7, 8^. Moreover, FXR promotes hepatic bile secretion by increasing the expression of the bile salt export pump (BSEP) and multidrug resistant protein 2 (MDR2) that transport BAs and phosphatidylcholine, respectively, to the canalicular lumen^9, 10^. In the intestine, FXR promotes enterocyte reabsorption and recycling of BAs to the liver. Importantly, BA-bound FXR induces ileal expression of the fibroblast growth factor 15/19 (*Fgf15/19*) in mice and humans, respectively. FGF15/19 is a hormone subsequently secreted in the portal vein that travels back to the liver where, in synergy with SHP, activates a signaling cascade that ultimately repress CYP7A1 expression^11^.

The important role of FXR in repressing BA synthesis has been initially shown in Fxr null mice, which exhibit an increased BA pool size and display increased expression of pro-inflammatory cytokines, resistance to apoptosis and cell hyperproliferation leading to development of spontaneous HCC between 12 and 15 months of age^12–16^.

We have previously shown that the intestinal specific iVP16FXR transgenic mouse model is able to protect mice from chemically- and genetically-induced cholestasis through downregulation of BA synthesis and upregulation of intestinal BA disposal^4^. More recently, we have provided compelling evidence that intestinal FXR is sufficient to protect against hepatocarcinogenesis by limiting BA overload, restoring the *Fgf15/Fgfr4* enterohepatic signaling axis and upregulating BA detoxification and efflux pathways. Moreover, intestinal-specific FXR activation also protects the liver by reducing inflammation, hyperproliferation and collagen deposition^3^. These studies in transgenic models clearly show that FXR represses hepatic inflammation and cell hyperproliferation, indicating that therapeutic modulation of FXR could be beneficial in patients with liver carcinoma.

Abcb4^−/−^ mice are a model of chronic cholangiopathy. Previously, it has been shown in this model that INT-767 is able to reduces BA toxicity by decreasing biliary BA output in an FXR-dependent fashion, resulting in the repression of hepatic inflammation and fibrosis^17^. Abcb4^−/−^ mice also represent a model of inflammation-associated HCC^18^. They lack the liver-specific P-glycoprotein responsible for phosphatidylcholine transport across the bile canalicular membrane, and the absence of phospholipids in bile results in bile regurgitation into the portal tracts^19^, causing portal inflammation and fibrosis that mimic human progressive familial intrahepatic cholestasis^20^. Liver inflammation and toxicity induced by BAs in Abcb4^−/−^ mice lead to hepatocyte dysplasia, evolving in HCC at 12–15 months of age.

In the present work, we show that long-term oral administration of the dual FXR/ membrane G protein-coupled receptor (TGR5) agonist INT-767 prevents HCC formation in Abcb4^−/−^ and not in Fxr^−/−^ mice. Strikingly, INT-767 administration in Abcb4^−/−^ mice stimulates intestinal *Fgf15* induction and inhibits hepatic *Cyp7a1* expression, promoting endogenous BA reduction, indicating that protection from HCC development is mediated by FXR and not TGR5 and underscoring the potential of FXR activation in disease with BA-associated HCC development.

## Results

### INT-767 prevents spontaneous HCC development in Abcb4^−/−^ but not in Fxr^−/−^ mice

**Figure 1:**
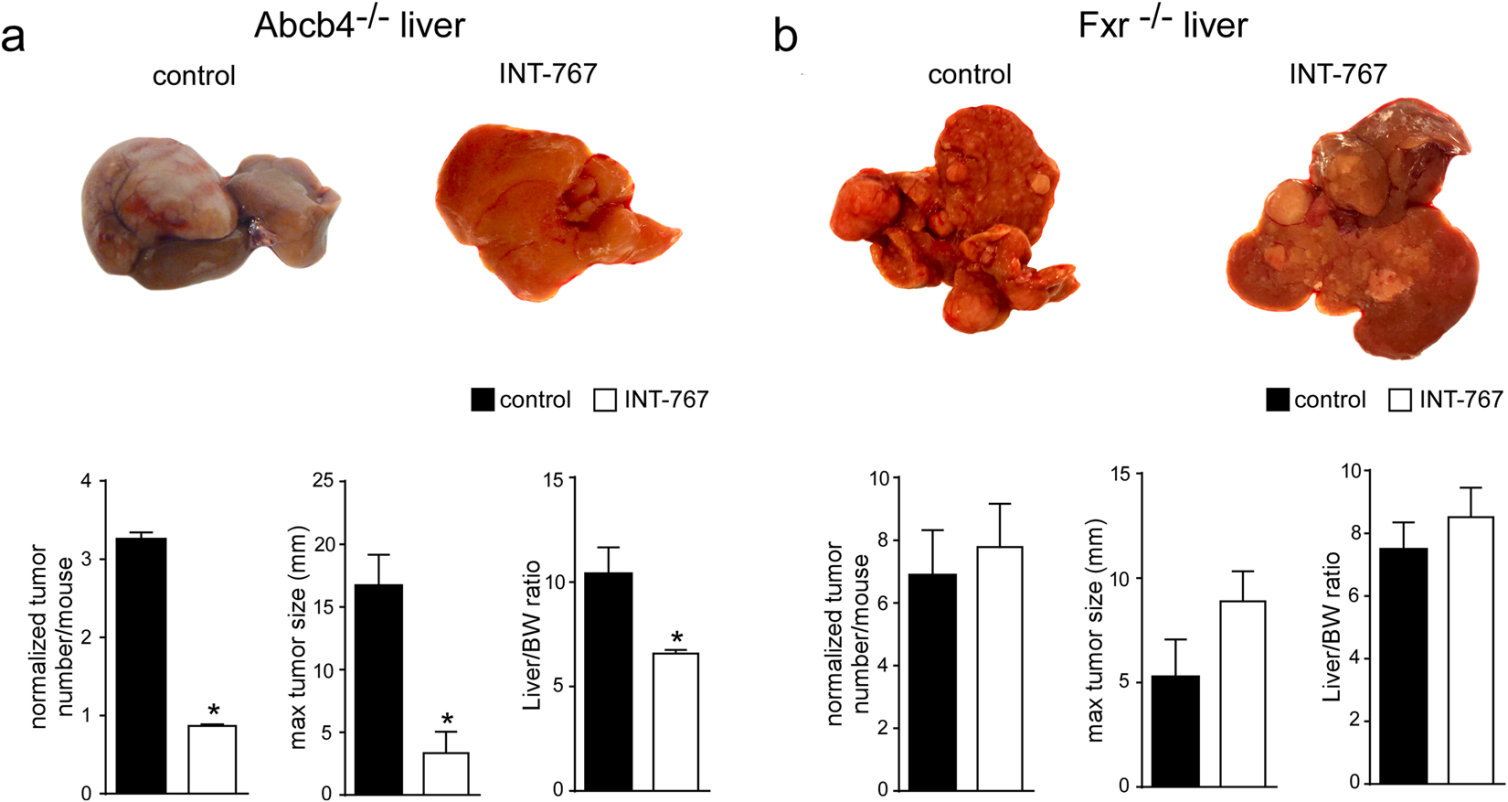
Prevention of spontaneous hepatocarcinogenesis in Abcb4^−/−^ mice by long-term administration of INT-767. Abcb4^−/−^ and Fxr^−/−^ mice were randomly divided into 2 experimental groups and fed with specific rodent diet containing 62.5 mg/Kg of INT-767 and control diet for 15 months. (**a**) Gross morphology of Abcb4^−/−^ INT-767-fed mice and controls. Normalized tumor number/mouse, maximal tumor sizes (mm) and body/liver weight ratio were reported. (**b**) Gross morphology of Fxr^−/−^ INT-767-fed mice and controls. Normalized tumor number/mouse, maximal tumor sizes (mm) and body/liver weight ratio were reported. The results are expressed as mean ± SEM. Statistical significance (P < 0.05) was assessed by Mann-Whitney’s U test.

To study the effects of INT-767 on HCC prevention, we administered either INT-767-enriched or vehicle diet to Abcb4^−/−^ and Fxr^−/−^ mice for 15 months. Our findings revealed that long-term administration of INT-767 in Abcb4^−/−^ mice exerts broad hepatoprotective effects, prompting analysis of HCC prevention. As shown in Fig. 1a, Abcb4^−/−^ control mice displayed grossly identifiable liver tumors while no or only very small tumors were found in INT-767-fed mice, demonstrating that INT-767 prevents HCC development in this model. In contrast, no changes were observed in liver tumor number and size in Fxr^−/−^ mice fed with or without INT-767 (Fig. 1b). In agreement with these data, a significant reduction in LW/BW ratio was found only in Abcb4^−/−^ INT-767 mice (Fig. 1). These data clearly indicate that long-term administration of INT-767 prevents HCC development in Abcb4^−/−^ mice in an FXR-dependent fashion.

### INT-767 improves hepatic function in Abcb4^−/−^ mice

**Figure 2:**
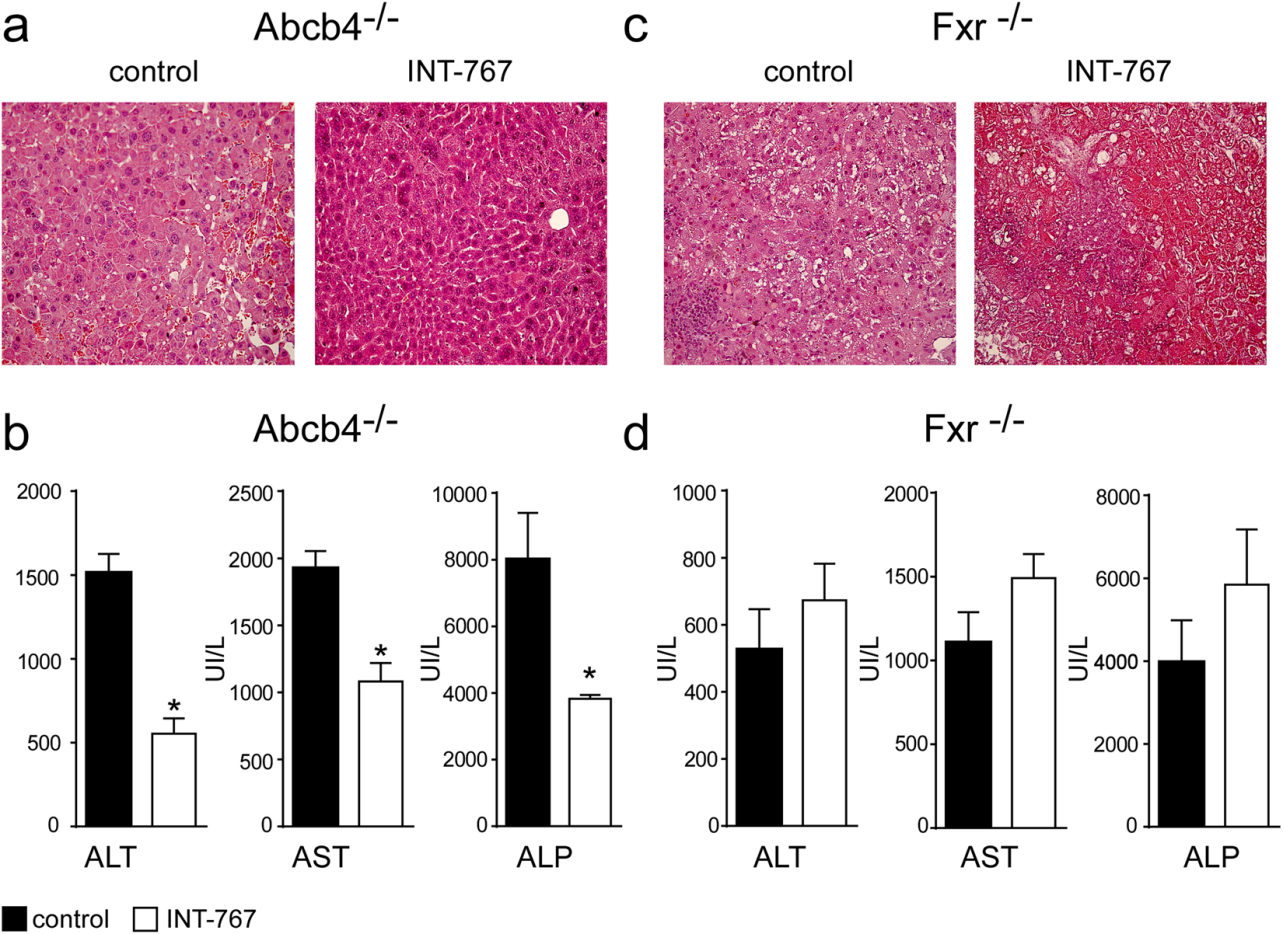
Long-term administration of INT-767 promotes endogenous BA reduction and reduces liver damage. (**a**) Liver histology was assessed by (H&E) staining and was observed by light microscopy (magnification, 200X) in Abcb4^−/−^ and (**c**) Fxr^−/−^ mice fed with or without INT-767. Representative specimens are shown. (**b**) Biochemical parameters of liver damage in Abcb4^−/−^ and (**d**) Fxr^−/−^ mice fed with or without INT-767. The results are expressed as mean ± SEM. Statistical significance (P < 0.05) was assessed by Mann-Whitney’s U test.

In order to evaluate the effects of INT-767 administration in Abcb4^−/−^ and Fxr^−/−^ mice we analyzed liver structure and function. Histological examination (H&E) of Abcb4^−/−^ INT-767-fed mice livers displayed a more preserved hepatic parenchyma than control mice (Fig. 2a) whereas a disrupted hepatic structure with prominent cell necrosis was detected in INT-767-treated Fxr^−/−^ mice compared to controls (Fig. 2c). In Abcb4^−/−^ but not in Fxr^−/−^ INT-767-fed mice, liver histological analysis revealed hepatoprotection as confirmed by the significant reduction of liver enzymes alanine aminotransferase (ALT), aspartate aminotransferase (AST), and alkaline phosphatase (ALP) compared to vehicle diet fed mice (Fig. 2b–d).

### INT-767 decreases hepatic expression of pro-inflammatory genes

**Figure 3:**
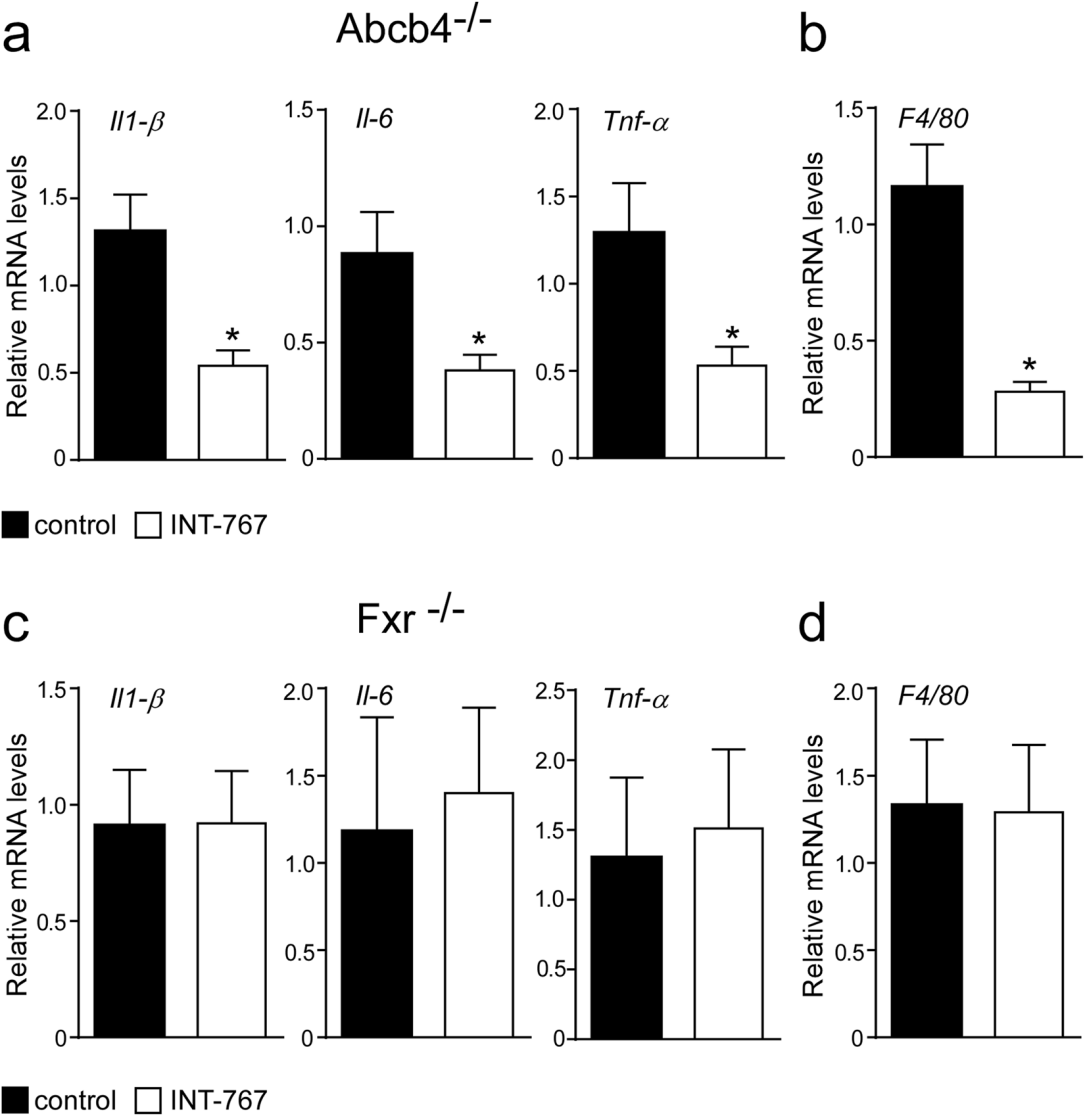
Prevention of hepatic inflammation by long-term administration of INT-767. (**a**) Gene expression analysis of inflammatory cytokines and (**b**) macrophage markers in Abcb4^−/−^ INT-767-fed mice and controls. (**c**) Gene expression analysis of inflammatory cytokines and (**d**) macrophage markers in Fxr^−/−^ INT-767-fed mice and controls. Cyclophilin was used as a housekeeping gene to normalize data. The results are expressed as mean ± SEM. Statistical significance (P < 0.05) was assessed by Mann-Whitney’s U test.

Macrophage and monocyte populations are an important source of cytokines in the liver and play a key role in liver homeostasis^21^. FXR activation reduced hepatic macrophages infiltration in Abcb4^−/−^ INT-767-fed mice compared to controls (Fig. 2a) as confirmed by a significant reduction of F4/80 expression^22^ (Fig. 3b). In Abcb4^−/−^ mice, INT-767 treatment reduced the expression of the pro-inflammatory cytokines *Il-1β*, *Il-6* and *Tnf-α* (Fig. 3a). Conversely, INT-767 administration was unable to modify macrophage infiltration and cytokine expression in Fxr^−/−^ mice (Fig. 3c,d). Taken together, these data indicate that INT-767 promotes via FXR activation an anti-inflammatory phenotype in Abcb4^−/−^ mice.

### INT-767 prevents liver proliferation and fibrosis

**Figure 4:**
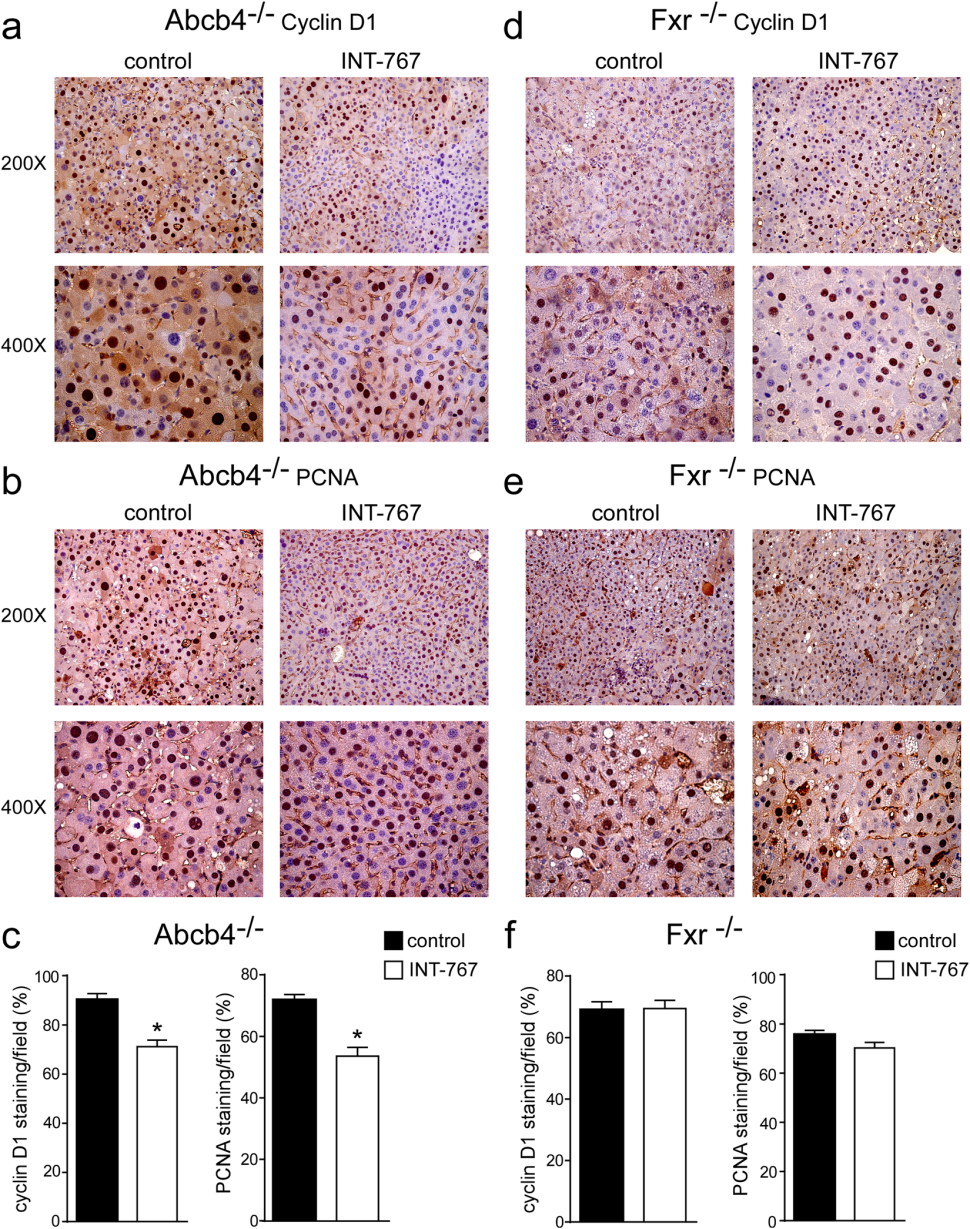
Prevention of hepatic proliferation by long-term administration of INT-767. (**a**) Paraffin-embedded liver specimens from Abcb4^−/−^ and (**d**) Fxr^−/−^ mice fed with or without INT-767 were immunoassayed with cyclin D1 antibody (200X and 400X magnification). Representative specimens are shown. (**b**) Paraffin-embedded liver specimens from Abcb4^−/−^ and (**e**) Fxr^−/−^ mice fed with or without INT-767 were immunoassayed with anti-PCNA antibody (200x and 400x magnification). Representative specimens are shown. (**c**) Cyclin D1 and PCNA staining per field was quantified by ImageJ software and reported as percentage per field. Comparison of Abcb4^−/−^ INT-767-fed mice and controls (n = 6) was performed using Mann-Whitney U test. Results are expressed as mean ± SEM (P < 0.05). (**f**) Cyclin D1 and PCNA staining per field was quantified by ImageJ software and reported as percentage per field. Comparison of Fxr^−/−^ INT-767-fed mice and controls (n = 6) was performed using Mann-Whitney U test. Results are expressed as mean ± SEM (P < 0.05).

**Figure 5:**
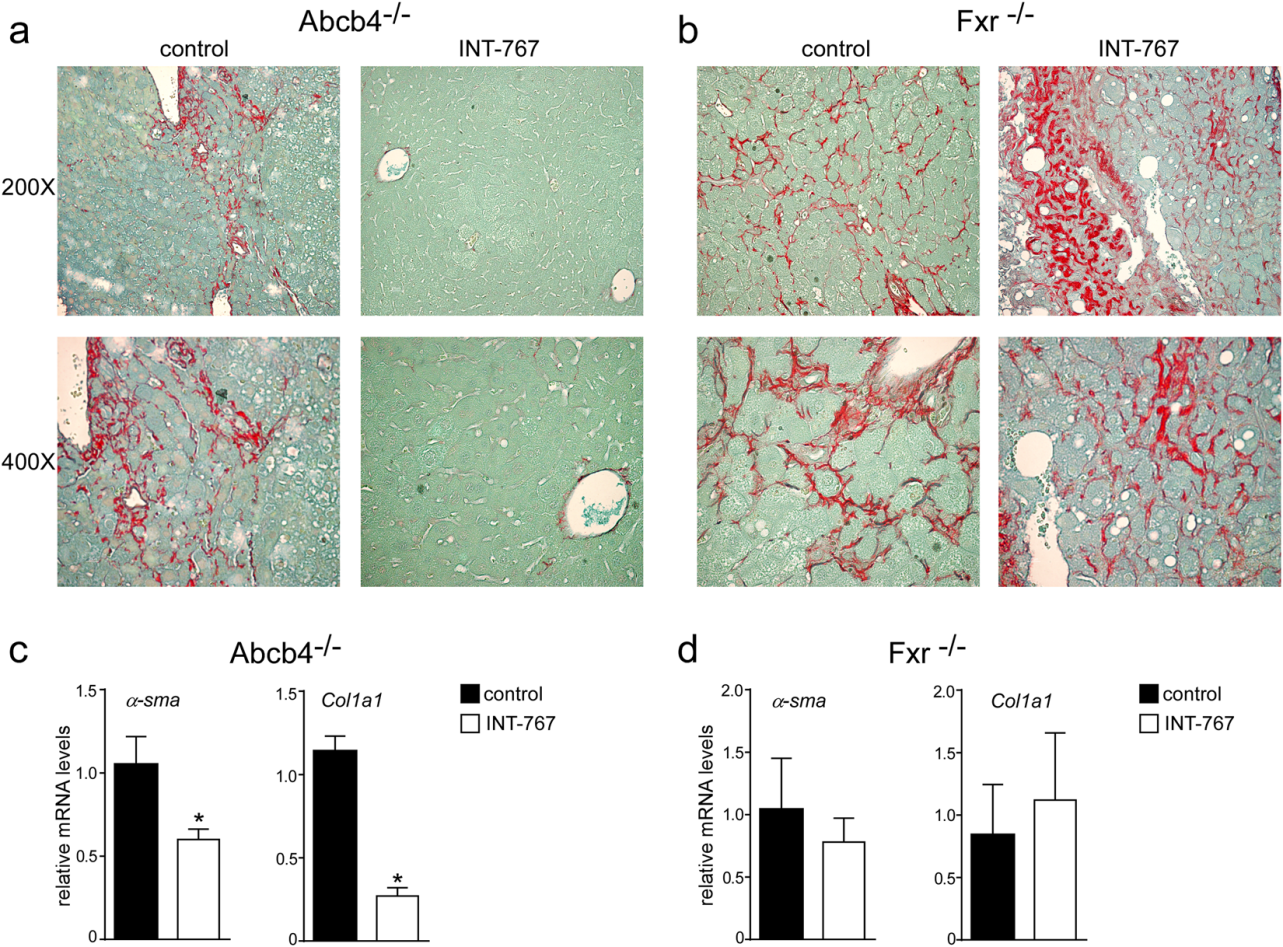
Prevention of hepatic fibrosis by long-term administration of INT-767. (**a**) Paraffin-embedded liver specimens from Abcb4^−/−^ and (**b**) Fxr^−/−^ mice fed with or without INT-767 were stained with Sirius Red and observed by light microscopy (200X and 400X magnification). Representative specimens are shown. (**c**) Gene expression analysis of fibrosis markers in Abcb4^−/−^ INT-767-fed mice and controls. (**d**) Gene expression analysis of fibrosis markers in Fxr^−/−^ INT-767-fed mice and controls. Cyclophilin was used as a housekeeping gene to normalize data. The results are expressed as mean ± SEM. Statistical significance (P < 0.05) was assessed by Mann-Whitney’s U test.

To explore the mechanisms underlying INT-767 mediated hepatoprotection, we examined the alterations of cell cycle. Proliferating cell nuclear antigen (PCNA) and CyclinD1 are regulators of cell cycle progression, and hepatic Shp over-expression has been associated with lower CyclinD1 levels^23^. In agreement with previous data, Fxr activation significantly reduced cyclinD1 and PCNA protein accumulations in Abcb4^−/−^ INT-767-fed mice compared to vehicle diet fed mice (Fig. 4a–c), whereas no difference was shown in Fxr^−/−^ mice (Fig. 4d–f). Furthermore, we evaluated the extent of collagen deposition in livers isolated from Abcb4^−/−^ and Fxr^−/−^ mice, and we found less sirius red positive areas together with a significant reduction of fibrosis gene expression markers such as *α-Sma* and *Collagen1a1* (*Col1a1*) only in Abcb4^−/−^ INT-767-fed mice (Fig. 5a–d). These data indicate that INT-767 displays an anti-proliferative effect and counteracts liver fibrosis via FXR/SHP activation.

### INT-767 reduces bile acid synthesis through Fxr/Fgf15 activation

**Figure 6:**
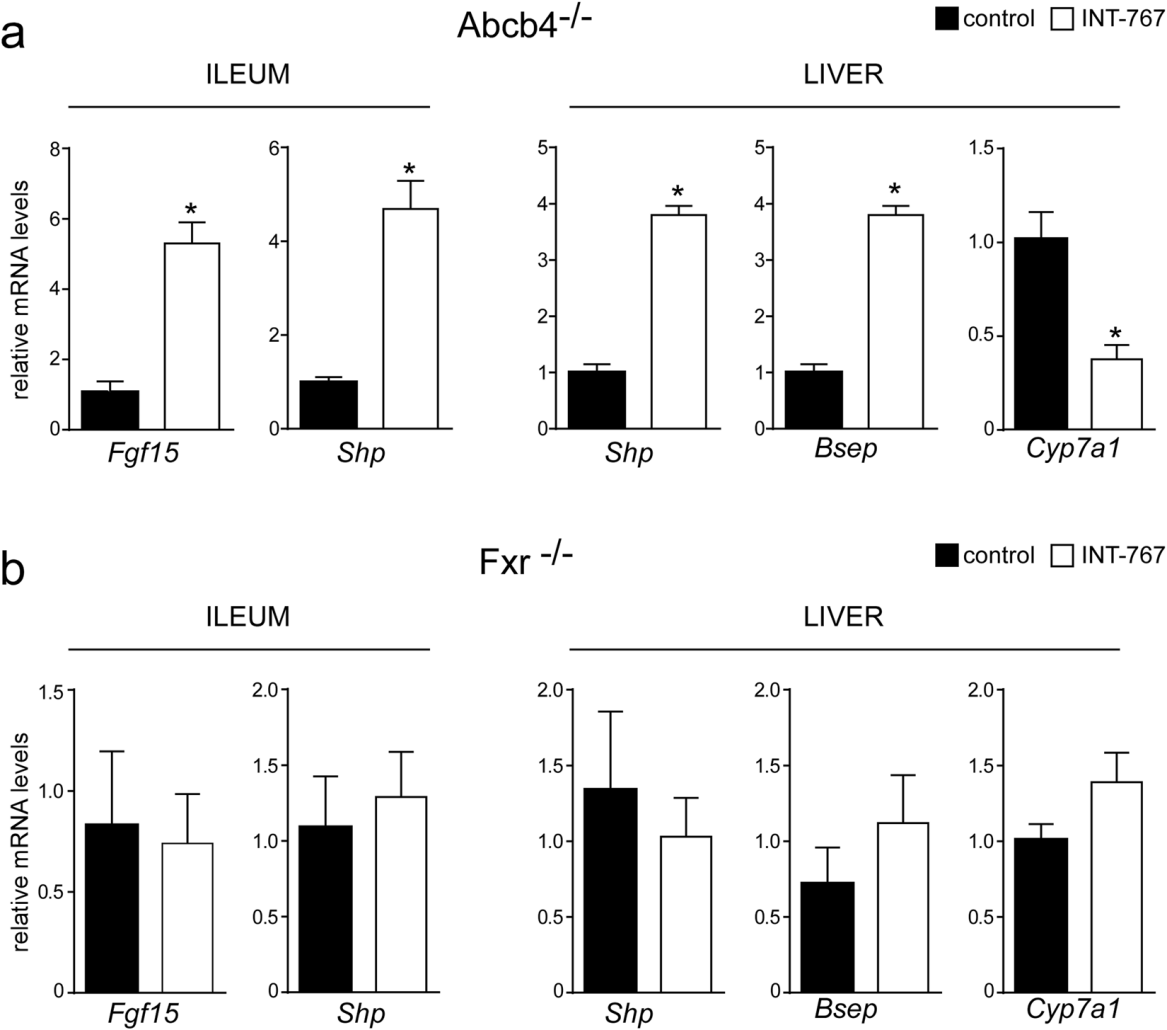
Gene expression analysis of FXR-regulated genes (**a**) in Abcb4^−/−^ and (**b**) Fxr^−/−^ mice fed with or without INT-767. Gene expression analysis of ileum and liver are reported. Cyclophilin was used as a housekeeping gene to normalize data and wild type mice as calibrators. (**c**) Serum BA levels in Abcb4^−/−^ and Fxr^−/−^ mice fed with or without INT-767. The results are expressed as mean ± SEM. Statistical significance (P < 0.05) was assessed by Mann-Whitney’s U test.

**Figure 7:**
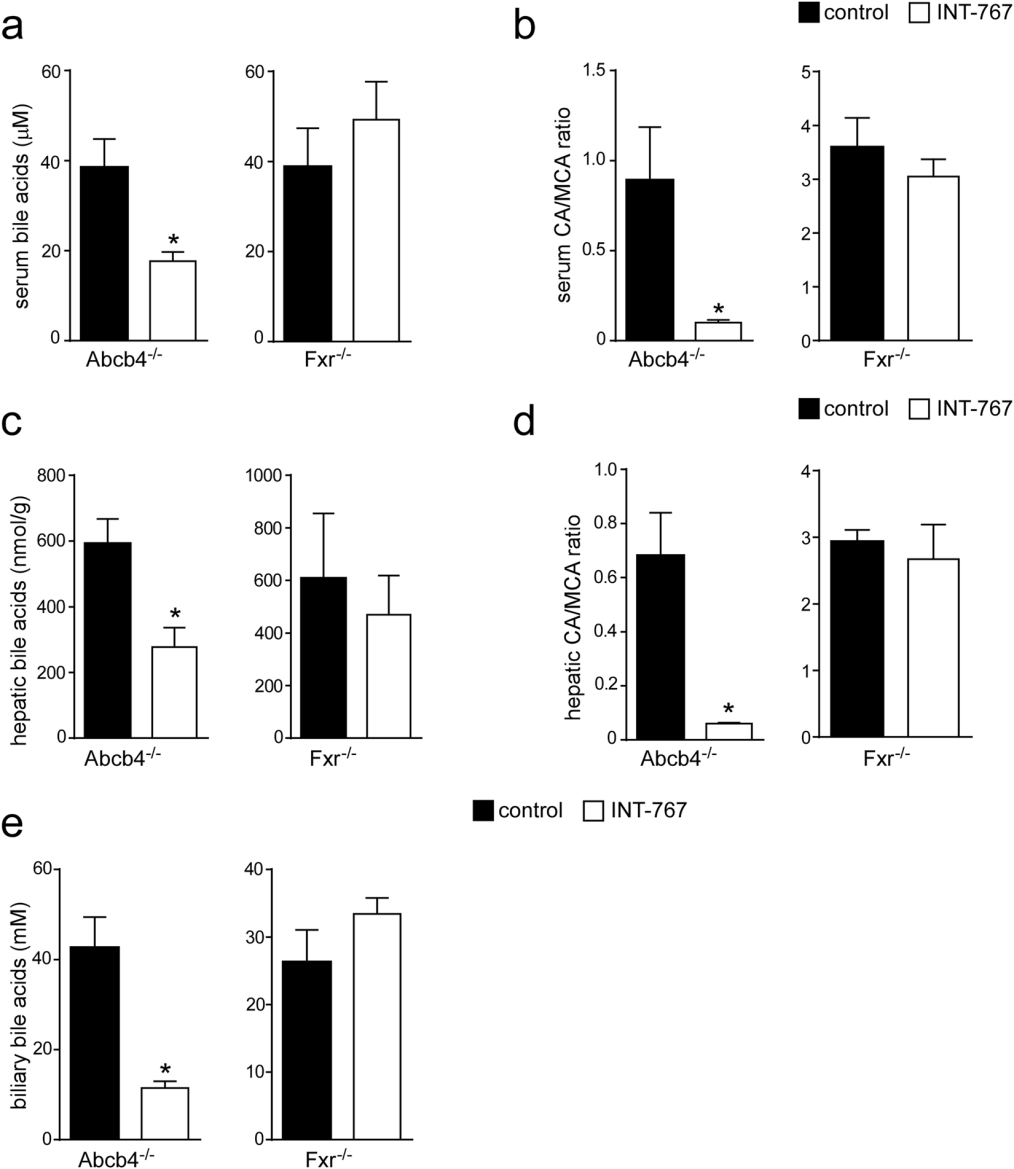
BAs pool size and composition in Abcb4^−/−^ and Fxr^−/−^ mice fed with or without INT-767. **(a)** Serum, **(c)** hepatic and **(e)** biliary BA levels in Abcb4^−/−^ and Fxr^−/−^ mice fed with or without INT-767. **(b)** Serum and **(d)** hepatic BA composition in Abcb4^−/−^ and Fxr^−/−^ mice fed with or without INT-767 is expressed as CA/MCA ratio. The results are expressed as mean ± SEM. Statistical significance (P < 0.05) was assessed by Mann-Whitney’s U test.

To study the effects of INT-767 on FXR transcriptional activation in the gut-liver axis, we analyzed hepatic and intestinal gene expression of Abcb4^−/−^ INT-767-fed mice. Data revealed a significant up-regulation of FXR target genes, including fibroblast growth factor 15 (*Fgf15*) and small heterodimer partner (*Shp*) leading to gut-liver synergistic inhibition of BA synthesis, as indicated by *Cyp7a1* down-regulation (Fig. 6a). No difference was found in Fxr^−/−^ mice treated with INT-767 compared to control fed mice (Fig. 6a). Moreover, in Abcb4^−/−^ INT-767-fed mice the inhibition of *Cyp7a1* was associated with a significant reduction of serum, hepatic and biliary BA levels (Fig. 7a–c–e). In these mice we observed a shift in both serum and liver BA composition to a more hydrophilic BA pool profile due to the enrichment in beta-muricholic acid (MCA) (Fig. 7b–d). The central role of FXR in the INT-767 effect was further confirmed by the observation that no reduction of BA levels or modification in their composition occurred in Fxr^−/−^ mice (Fig. 7).

## Discussion

In this study, we have addressed the role of long-term FXR activation in the pathogenesis of HCC by orally administering INT-767 to Abcb4^−/−^ and Fxr^−/−^ mice, two mouse models of spontaneous liver tumorigenesis. We report herein that only in Abcb4^−/−^ mice INT-767 is able to reduce bile toxicity by decreasing total BA output in a FXR-dependent manner, thus preventing BA-induced tumor promoting effects^24^.

Deregulation of BAs leads to several pathological consequences, such as cholestasis and cancer, and abnormal BA levels resulting from disruption of metabolic homeostasis have been observed to increase cell proliferation in the liver and intestine^24–26^. Abnormally high concentrations of BAs induce cell death and inflammation, thereby promoting cancer development^25^, and the association of BAs with liver cancer has been demonstrated in both human and animal studies^12, 13, 27, 28^. Knisely *et al*. reported a link between progressive familial intrahepatic cholestasis type 2 and HCC in children with cholestasis^29^. Similarly, mice with *Abcb4* gene deletion present BA accumulation inside the liver, with progressive development of inflammation, HCC and cholangiocarcinoma^30^.

FXR is the master transcriptional regulator of BA homeostasis, controlling BA synthesis, influx, efflux, and detoxification in the gut/liver axis. FXR has been implicated in liver tumorigenesis^31–33^. Fxr^−/−^ mice present increase BA pool size and spontaneously develop with age liver tumors^12, 13^. Feeding Fxr^−/−^ mice with a cholic acid diet further promoted chemical-induced hepatocarcinogenesis^13^. We have previously shown that in the transgenic tissue-specific mouse model (iVP16FXR) FXR activation protects mice from cholestasis by reducing BA synthesis and promoting intestinal BA disposal^4^. Furthermore, intestinal FXR activation prevents BA overload and hepatocarcinogenesis through the restoration of FXR-Fgf15-Cyp7a1 axis^3^. Thus, this pathway provides promising targets for pharmacological intervention in HCC.

INT-767 is a semisynthetic 23-sulfate derivative of obeticholic acid (OCA), the FXR agonist recently approved for the treatment of patients with primary biliary cholangitis. INT-767 differs from OCA in that it is a more potent agonist for FXR, with a EC50 of 30 nM. Moreover, at a variance of OCA, INT-767 is also an affective agonist for TGR5 (also called GPBAR1 or M-BAR/BG37)^34^, with a EC50 of 630 nM. Thus, INT-767 is the first compound to potently and selectively activate both BA receptors^17, 34^. Our results show that in Abcb4^−/−^ mice long-term administration of INT-767 induces FXR activation that in turn increases *Fgf15* expression, triggering a synergistic gut-liver signaling pathway involving *Shp* and hepatic *Cyp7a1* to down-regulate total BAs. In addition, INT-767 *via* FXR activation exhibits hepatoprotective effects in the Abcb4^−/−^ model, as shown by reduced serum hepatic enzyme levels and a preserved tissue morphology. FXR is highly expressed in hepatocytes and cholangiocytes, whereas TGR5 is mainly expressed in Kupffer cells within the liver^35^. TGR5 is a key receptor for mediating the effects of BAs in regulating energy metabolism, insulin signaling, and inflammatory response. High levels of TGR5 are also detected in gallbladder epithelial cells, gallbladder smooth muscle cells, and sinusoidal endothelial cells^35^. Our data show that INT-767 has no effects on HCC development in Fxr^−/−^ mice, indicating that protection from spontaneous hepatocarcinogenesis in Abcb4^−/−^ mice is solely mediated by FXR activation and does not appear to involve TGR5.

In db/db mice, INT-767 improved hepatic histological features, recruiting Ly6C^Low^ monocytes to the liver and decreasing pro-inflammatory cytokines production by macrophages^22^. Overexpression of FXR suppresses the expression of inflammatory mediators in both HepG2 cells and primary hepatocytes, while Fxr^−/−^ mice showed increased levels of several pro-inflammatory cytokines in response to lipopolysaccharide (LPS) stimuli^36, 37^. In agreement with these results, in Abcb4^−/−^ INT-767 fed mice, FXR activation reduced macrophages liver infiltration and the hepatic expression of pro-inflammatory cytokines *Il-1β*, *Il-6* and *Tnf-α*. Therefore, INT-767 administration in Abcb4^−/−^ mice conferred hepatoprotection through reduced inflammation as well as limited collagen deposition, finally resulting in decreased liver injury, suggesting that INT-767 acts as a specific FXR modulator able to suppress liver inflammation and fibrosis. Thus, we provide evidence that long-term administration of INT-767 through FXR activation prevents spontaneous HCC development by controlling BA synthesis through *Cyp7a1* reduction, limiting hepatic inflammation, proliferation, and fibrosis, consistent with the capacity of FXR to confer hepatoprotection and suppress HCC by controlling BA homeostasis^12, 13, 38^ and preventing hepatic inflammation and fibrosis^37^. In addition, FXR activates the expression of Shp, which also contrasts HCC development^38^.

In conclusion, our study demonstrates that in Abcb4^−/−^ mice FXR activation by long-term administration of INT-767 stimulates FGF15 production, thereby repressing hepatic *Cyp7a1* expression, and ultimately leading to endogenous BA reduction and to HCC prevention. HCC is among the most lethal and prevalent human tumors but despite its relevance, only limited therapeutic options, mostly with negligible clinical benefit, are available^39, 40^. We believe that our findings on INT-767 induced prevention of HCC will raise the interest in addressing the role of FXR agonist in the treatment of HCC both in preclinical and clinical settings.

## Methods

### Spontaneous hepatocarcinogenesis in 16-months-old Abcb4^−/−^ and Fxr^−/−^ mice

Pure strain C57BL/6 J Fxr^−/−^ mice were kindly provided by Dr. D. Mangelsdorf (Southwestern Medical Center, Dallas, TX) and were generated by backcrossing with C57BL/6 J mice for more than 15 generations the original Fxr^−/−^ mice (kindly provided by Dr. F. Gonzalez, NIH, Bethesda, MD). Pure strain FVBN/Abcb4^−/−^ mice were kindly provided by Dr A. K. Groen (Amsterdam, The Netherlands). For spontaneous hepatocarcinogenesis model, at one month of age twenty-eight male Abcb4^−/−^ and twenty male Fxr^−/−^ mice were randomly divided into 2 experimental groups and fed with specific rodent diet containing 62.5 mg/Kg of the dual FXR-TGR5 agonist INT-767, Intercept Pharmaceuticals Inc, NY) and control diet for 15 months. During the experimental period, individual body weight was recorded every 7 days. All mice were housed under pathogen-free conditions in a temperature-controlled room (23 °C) on a 12-hour light/dark cycle and fed specific or control diets and water *ad libitum*. After 15 months, mice were sacrificed and serum, liver and intestine were collected. The total number of hepatic tumors was counted and the diameter of each tumor was measured.

### Serum analysis

Levels of ALT, AST and ALP were measured with a colorimetric kit (BioQuant Heidelberg, Germany) according to manufacturer’s instructions.

### Chemicals

Cholic acid (CA) and other endogenous BAs were purchased from Sigma-Aldrich (St. Louis, MO). The molecules INT-767, its 3-glucuronide conjugates (Glucu-INT-767) and internal standard OCA, INT-747 were supplied by Intercept Pharmaceuticals, Inc. (ICPT). All solvents were of high purity and used without further purification. Acetonitrile for HPLC was from Merck (Darmstadt, Germany); methyl alcohol RPE, ammonia solution 30% RPE, glacial acetic acid RPE were from Carlo Erba Reagent (Milan, Italy); activated charcoal was from Sigma-Aldrich; and ISOLUTE C18 cartridges (500 mg, 6 ml) for the plasma sample pretreatment were purchased from StepBio (Bologna, Italy). Plasma BA free mouse plasma was treated with activated 50 mg/ml charcoal and stirred at 4 °C overnight. After centrifugation at 3000 g for 5 minutes the plasma was filtered through Millipore A10 Milli-Q Synthesis (0.45 µm) and stored at −20 °C.

### BA Measurements

Biliary bile acids were measured with a colorimetric kit (Diazyme, Poway, CA) according to manufacturer’s instructions. Serum and hepatic BAs were identified and quantified by high-pressure liquid chromatography-electrospray-mass spectrometry/mass spectrometry (HPLC-ES-MS/MS) by optimized methods^41^ suitable for use in pure standard solution, plasma and liver samples after appropriate clean-up preanalytical procedures. Liquid chromatography analysis was performed using an Alliance HPLC system model 2695 from Waters combined with a triple quadruple mass spectrometer QUATTRO-LC (Micromass; Waters) using an electrospray interface. The analytical column was a Waters XSelect CSH Phenyl-hexyl column, 5 µm, 150 × 2.1 mm, protected by a self-guard column Waters XSelect CSH Phenyl- hexyl 5 µm, 10 × 2.1 mm. BAs were separated by elution gradient mode with a mobile phase composed of a mixture ammonium acetate buffer 15 mM, pH 8.0 (Solvent A) and acetonitrile:methanol = 75:25 v/v (Solvent B). Chromatograms were acquired using the mass spectrometer in multiple reaction monitoring mode.

### Serum Extraction Method

100-µL aliquots of serum samples were fortified with 10 µL of the internal standard working solution and were diluted by adding 2 mL of NaOH 0.1 N and heated to 64 °C for 30 minutes. The solid phase extraction (SPE) cartridge was conditioned with 5 mL of methanol and 5 mL of water prior to sample loading. Serum samples were loaded onto the conditioned cartridge and then washed with 10 mL of water. The cartridge was then eluted with 5 mL of methanol and the eluate was collected. The eluate was dried under vacuum and then reconstituted with 100 µL of the mobile phase (65:35 v/v = ammonium acetate buffer 15 mM pH = 8.0: (acetonitrile:methanol = 75:25 v/v), and injected into the HPLC-ES-MS system.

### Liver Extraction Method

Aliquots weighing approximately 0.2–0.3 g each were taken from different points of the liver sample. Each aliquot was exactly weighed, and 1 mL of phosphate buffer (0.005 M, pH 7.2) was added. The mixture was homogenized using a potter, and the potter was washed with methanol (3 × 0.5 mL). The mixture was sonicated for 5 minutes, vortexed for 2 minutes, heated to 37 °C for 20 minutes, and centrifuged at 2100 x g for 15 minutes. 200 µL of the supernatant was spiked with 10 µL of the internal standard working solution and was dried under vacuum. Then, the residue was resuspended with 2 mL of sodium hydroxide (0.1 N). The solution was sonicated for 10 minutes, heated to 64 °C for 30 minutes, and SPE was carried out on C18 extraction cartridges. The SPE cartridge was conditioned with 5 mL of methanol and 5 mL of water prior to sample loading. Then, the liver sample extract of 2 mL was loaded onto the conditioned cartridge and washed with 10 mL of water. The cartridge was then eluted with 5 mL of methanol. The eluate was dried under vacuum and reconstituted with 100 µL of the mobile phase (65:35 v/v = ammonium acetate buffer 15 mM pH = 8.0: (acetonitrile:methanol = 75:25 v/v), and injected into the HPLC-ES-MS system. For each BA, stock solutions were prepared in methanol at 1 mM and stored at approximately −20 °C. Stock solutions were further diluted with methanol to obtain working solutions containing all the BAs studied. A six-point calibration curve (0.1 to 20 μM) was prepared by adding the appropriate amount of each corresponding bile acid working solution and suitable volume of internal standard working solution to obtain a concentration of 1 µM. For plasma calibration curve, plasma free was used to quantify plasma samples with clean-up following SPE procedure. Linear calibration curve parameters were obtained from the plot of the analyte peak area/internal standard peak area versus analyte concentration using a least squares regression analysis (weight = 1/x). Then, absolute µmoles were calculated to express the final results. When concentration samples were out of linear range, they were submitted to re-analysis, performing an appropriate dilution. In terms of linearity, accuracy, and precision, the analytical method used in this study fulfils the compliance criteria described by the Food and Drug Administration guidance for the industry: bioanalytical method validation. Limits of detection (LOD) and limits of quantification (LOQ), estimated as the signal-to-noise ratio (S/N) equal respectively to 3 and 9, were for each bile acid of no more than 20 nM for each analyte.

### RNA Extraction

Total RNA was isolated by Qiazol reagent (Qiagen) following the manufacturer’s instructions. To avoid possible DNA contaminations, RNA was treated with DNase I (Ambion). RNA purity was checked by spectrophotometer and RNA integrity by examination on agarose gel electrophoresis. cDNA was synthesized retrotranscribing 4 μg of total RNA in a total volume of 100 μL using a High Capacity DNA Archive Kit (Applied Biosystems) for the manufacturer’s instructions.

### Real-time quantitative PCR

Real-time quantitative PCR (RTqPCR) primers were designed using Primer Express software. PCR assays were performed in 96-well optical reaction plates using the QuantStudio5 machine (Thermo Fisher Scientific). PCR assays were conducted in triplicate wells for each sample. Baseline values of amplification plots were set automatically, and threshold values were kept constant to obtain normalized cycle times and linear regression data. The reaction mixture per well used were as follows: 10 μl Power Syber Green (Thermo Fisher Scientific), 2.4 μl of primers at the final concentration of 150 nmol/L, 4.6 μl RNAase free water, and 3 μl cDNA (60 ng). For all experiments, PCR conditions used were as follows: denaturation at 95 °C for 10 min, followed by 40 cycles at 95 °C for 15 s, then at 60 °C for 60 s. Quantitative normalization of cDNA in each sample was performed using cyclophilin as internal control. Relative quantification was performed using the ΔΔCT method. Validated primers for RTqPCR are available upon request.

### Histology and immunohistochemistry

Tissue specimens were fixed in 10% formalin for 12 to 24 h, dehydrated, and paraffin embedded. 4 µmn thick sections were stained with (H&E) following standard protocols. Liver fibrosis was analyzed with Sirius Red by using Direct Red 80 and Fast Green FCF (Sigma Aldrich, Milan, Italy). Briefly, sections were subjected to antigen retrieval by boiling the slides in sodium citrate pH 6 (Sigma Aldrich, Milan, Italy) for 15 min. Sections were permeabilized in phosphate-buffered saline (PBS) with 0.25% TritonX-100 for 5 min and were sequentially incubated for 10 min at room temperature in protein blocking solution (Dako, Glostrup, Denmark) and overnight at 4 °C with the primary antibodies (anti-pcna, Santa Cruz Biotechnology, Santa Cruz, CA; or anti-cyclin D1, Abcam, Cambridge, UK). Sections were washed 15 min in PBS and incubated for 25 min at room temperature with DAKO real EnVision detection system Peroxidase/DAB^+^ (Dako, Glostrup, Denmark) according to manufacturer’s instruction. After washing in PBS, the peroxidase reaction was initiated by incubation with DAB (Dako, Glostrup, Denmark). Coverslips were mounted with Permount and evaluated under a light microscope. Image processing was performed using Image J software. For each sample, 10 representative images were taken with a 20x objective. The percentage of stained area/total area was measured. Values from all consecutive images for each samples were averaged. For negative controls, 1% nonimmune serum in PBS replaced the primary antibodies.

### Statistical analysis

All results are expressed as mean ± SEM. Data distribution and gene expression statistical analysis were performed using GraphPad Prism software (v5.0; GraphPad Software Inc., San Diego, CA). Comparisons of two groups were performed using a Mann-Whitney’s U test. A p value of < 0.05 was considered significant.

### Ethics Statement

The Ethical Committee of the University of Bari approved this experimental set-up, which also was certified by the Italian Ministry of Health in accordance with internationally accepted guidelines for animal care.
